# Ultrafast structural changes direct the first molecular events of vision

**DOI:** 10.1038/s41586-023-05863-6

**Published:** 2023-03-22

**Authors:** Thomas Gruhl, Tobias Weinert, Matthew J. Rodrigues, Christopher J. Milne, Giorgia Ortolani, Karol Nass, Eriko Nango, Saumik Sen, Philip J. M. Johnson, Claudio Cirelli, Antonia Furrer, Sandra Mous, Petr Skopintsev, Daniel James, Florian Dworkowski, Petra Båth, Demet Kekilli, Dmitry Ozerov, Rie Tanaka, Hannah Glover, Camila Bacellar, Steffen Brünle, Cecilia M. Casadei, Azeglio D. Diethelm, Dardan Gashi, Guillaume Gotthard, Ramon Guixà-González, Yasumasa Joti, Victoria Kabanova, Gregor Knopp, Elena Lesca, Pikyee Ma, Isabelle Martiel, Jonas Mühle, Shigeki Owada, Filip Pamula, Daniel Sarabi, Oliver Tejero, Ching-Ju Tsai, Niranjan Varma, Anna Wach, Sébastien Boutet, Kensuke Tono, Przemyslaw Nogly, Xavier Deupi, So Iwata, Richard Neutze, Jörg Standfuss, Gebhard Schertler, Valerie Panneels

**Affiliations:** 1 Division of Biology and Chemistry, Laboratory for Biomolecular Research, Paul Scherrer Institute, Villigen PSI, Switzerland; 2 Photon Science Division, Laboratory for Femtochemistry, Paul Scherrer Institute, Villigen PSI, Switzerland; 3 Department of Chemistry and Molecular Biology, University of Gothenburg, Gothenburg, Sweden; 4 Institute of Multidisciplinary Research for Advanced Materials, Tohoku University, Sendai, Japan; 5 RIKEN SPring-8 Center, Hyogo, Japan; 6 Condensed Matter Theory Group, Laboratory for Theoretical and Computational Physics, Division of Scientific Computing, Theory and Data, Paul Scherrer Institute, Villigen PSI, Switzerland; 7 Swiss Institute of Bioinformatics (SIB), Lausanne, Switzerland; 8 Photon Science Division, Laboratory for Nonlinear Optics, Paul Scherrer Institute, Villigen PSI, Switzerland; 9 Institute of Molecular Biology and Biophysics, Department of Biology, ETH Zurich, Zurich, Switzerland; 10 Photon Science Division, Laboratory for Macromolecules and Bioimaging, Paul Scherrer Institute, Villigen PSI, Switzerland; 11 Division Scientific Computing, Theory and Data, Paul Scherrer Institute, Villigen PSI, Switzerland; 12 Department of Cell Biology, Graduate School of Medicine, Kyoto University, Kyoto, Japan; 13 Department of Biology, ETH Zurich, Zurich, Switzerland; 14 Japan Synchrotron Radiation Research Institute, Hyogo, Japan; 15 Laboratory for Ultrafast X-ray Sciences, École Polytechnique Fédérale de Lausanne, Lausanne, Switzerland; 16 Institute of Nuclear Physics Polish Academy of Sciences, Kraców, Poland; 17 Operando X-ray Spectroscopy, Energy and Environment Division, Paul Scherrer Institute, Villigen PSI, Switzerland; 18 Linac Coherent Light Source, SLAC National Accelerator Laboratory, Menlo Park, CA USA; 19 Present Address: European XFEL, Schenefeld, Germany; 20 Present Address: Biologics Center, Novartis Institutes for Biomedical Research, Basel, Switzerland; 21 Present Address: Linac Coherent Light Source, SLAC National Accelerator Laboratory, Menlo Park, CA USA; 22 Present Address: California Institute for Quantitative Biosciences (QB3), University of California, Berkeley, CA USA; 23 Present Address: Department of Physics, Utah Valley University, Orem, UT USA; 24 Present Address: Leiden Institute of Chemistry, Leiden University, Leiden, The Netherlands; 25 Present Address: Department of Molecular Biology and Genetics, Aarhus University, Aarhus, Denmark; 26 Present Address: Dioscuri Center For Structural Dynamics of Receptors, Faculty of Biochemistry, Biophysics and Biotechnology, Jagiellonian University in Kraków, Kraków, Poland

**Keywords:** X-ray crystallography, Photobiology, Visual system

## Abstract

Vision is initiated by the rhodopsin family of light-sensitive G protein-coupled receptors (GPCRs)^1^. A photon is absorbed by the 11-*cis* retinal chromophore of rhodopsin, which isomerizes within 200 femtoseconds to the all-*trans* conformation^2^, thereby initiating the cellular signal transduction processes that ultimately lead to vision. However, the intramolecular mechanism by which the photoactivated retinal induces the activation events inside rhodopsin remains experimentally unclear. Here we use ultrafast time-resolved crystallography at room temperature^3^ to determine how an isomerized twisted all*-trans* retinal stores the photon energy that is required to initiate the protein conformational changes associated with the formation of the G protein-binding signalling state. The distorted retinal at a 1-ps time delay after photoactivation has pulled away from half of its numerous interactions with its binding pocket, and the excess of the photon energy is released through an anisotropic protein breathing motion in the direction of the extracellular space. Notably, the very early structural motions in the protein side chains of rhodopsin appear in regions that are involved in later stages of the conserved class A GPCR activation mechanism. Our study sheds light on the earliest stages of vision in vertebrates and points to fundamental aspects of the molecular mechanisms of agonist-mediated GPCR activation.

## Abstract (web summary)

One picosecond after photoactivation, isomerized retinal pulls away from half of its numerous interactions with its binding pocket, and the excess of the photon energy is released through an anisotropic protein breathing motion in the direction of the extracellular space.

## Main

**Fig. 1:**
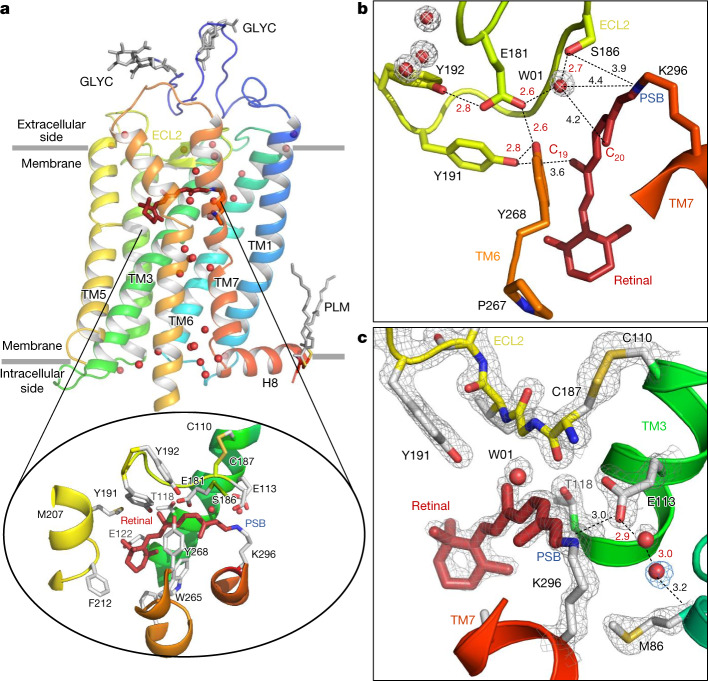
Room temperature SFX structure of the dark state of bovine rhodopsin from crystals grown in LCP. **a**, The overall structure of rhodopsin, rainbow coloured by residue number from blue (N terminus) to red (C terminus). The seven-TM bundle contains two *N*-glycosylation domains (GLYC) and palmitate groups (PLM) that anchor the amphipathic helix H8 to the membrane (grey lines). Water molecules (red spheres) form key networks^1^ between the extracellular (retinal ligand-binding pocket) and intracellular (G protein-binding site) regions of the receptor. The 11-*cis* retinal (dark red) is covalently bound to Lys296 (inset) through the PSB. The retinal-binding pocket is further composed of amino acids surrounding the PSB (the counterion Glu113, and Met44, Phe91, Thr94, Ala292 and Phe293), the retinal aliphatic chain (Ala117, Thr118, Tyr191, Trp265 and Glu181/Ser186 through water W01) and the β-ionone ring (Gly120, Gly121, Glu122, Phe212, Met207, Phe261 and Ala269; for clarity, only selected residues in the binding pocket are shown). **b**,**c**, Examples of well-resolved molecules in the water-mediated networks connecting the residues in the ancestral counterion Glu181 network (**b**) and the counterion Glu113 to Met86 of TM2 (and Ala117^3.32^, not shown) (**c**). The water molecules have well-defined electron densities (grey and blue meshes, 2*F*_obs_ − *F*_calc_ electron density contoured at 2.2 and 0.7*σ*, respectively).

Rhodopsin, the vertebrate receptor for low-light vision, is concentrated within the disk membranes of rod cells in the retina. Rhodopsin transforms the absorption of light into a physiological signal through conformational changes that activate the intracellular G protein transducin—a member of the Gi/o/t family—initiating a signalling cascade, resulting in electrical impulses sent to the brain and ultimately leading to visual perception. The structure of rhodopsin consists of seven transmembrane (TM) α-helices with an 11-*cis* retinal chromophore covalently bound through a protonated Schiff base (PSB) to Lys296^7.43^ of TM7 (the superscript values on amino acids containing the TM domain location refer to the Ballesteros–Weinstein scheme, explained in the ‘Residue numbering’ section of the Methods). This buried ligand is located within the TM bundle towards the extracellular side, like in many class A GPCRs. Retinal also contacts extracellular loop 2 (ECL2), which forms a lid over the chromophore and contains a highly conserved disulfide bridge (Cys110^3.25^–Cys187^ECL2^) connecting to the central helix TM3 (see box 1 of ref. ^1^). Glu113^3.28^ provides a negatively charged counterion^4^ that forms a salt bridge with the PSB (Fig. 1a–c) and thereby participates in the stabilization of the receptor resting state^5^. From our understanding of the evolution of visual pigments^6,7^, we know that, originally, Glu181^ECL2^ was the only residue able to neutralize the positive charge of the Schiff base. This ‘ancestral counterion’^8^, which still functions as a complex counterion in invertebrates^6^, remains connected through a water-mediated hydrogen bond network to the PSB of vertebrate rhodopsins (Fig. 1b). The second, main counterion Glu113^3.28^ appeared during evolution and both residues are important for the activation mechanism. Structures of light-activated rhodopsin trapped at low temperature^9,10^, structures of the late Meta II active state^11–13^, and copious computational^14^, biochemical and spectroscopic studies^15–17^ have provided important insights into the mechanism of signal transduction in rhodopsin. However, methods that provide both a high spatial and temporal resolution are required to obtain a complete experimentally derived picture of the activation mechanism at the atomic scale from femtoseconds to milliseconds.

In recent years, time-resolved crystallography^3,18^ at X-ray free-electron lasers (XFEL) has been used to reveal ultrafast structural changes in myoglobin^19^, photoactive yellow protein^20,21^, fluorescent proteins^22^, bacterial phytochromes^23^, microbial proton^24,25^, sodium^26^ and chloride^27^ pumps, a bacterial photosynthetic reaction centre^28^ and also with non-photosensory proteins with exogenously added non-natural photocaged ligands^29,30^. In time-resolved serial femtosecond crystallography (TR-SFX), the protein molecules in the crystals are photoactivated with an optical laser pulse and the structure is probed with an X-ray pulse from an XFEL after a specified time delay. As each crystal generates one diffraction pattern, the experiment is carried out in a serial manner: the frames are collected from tens of thousands of randomly oriented crystals. The TR-SFX method is complementary to spectroscopy methods, revealing structural detail at the atomic level in the femtosecond domain, without directly resolving charge effects, hydrogen bond interactions and electronic changes. The atomic resolution comes at the cost of less clearly defined illumination conditions^31^, which are a matter of discussion^24,25,32^, and systematic studies investigating these in more detail are underway^33^.

Here we used TR-SFX at room temperature to follow the light-induced conversion of the inverse agonist 11-*cis* retinal to an agonist all-*trans* in a vertebrate opsin. Our observations reveal how this translates into early structural changes within the protein. After 1 ps, we observe a twisted retinal that stores energy while structural motions in the protein radiate as an anisotropic propagation away from the retinal chromophore. The rhodopsin structure, 100 ps later, reveals a slightly more relaxed conformation.

## Room temperature structure of rhodopsin

**Extended Data Fig. 1:**
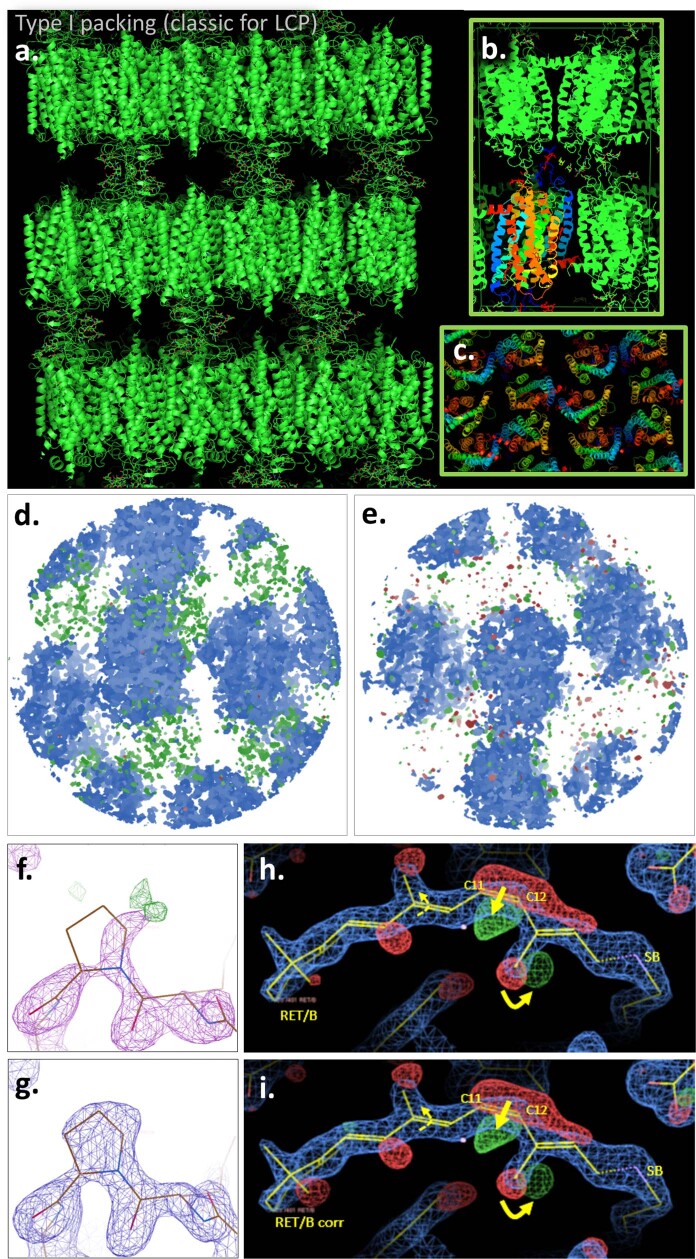
Rhodopsin molecules crystal packing and lattice translation correction. **a-c.)** Rhodopsin molecules packing in the crystal lattice of space group P 2 2_1_ 2_1_. Bovine rhodopsin crystals obtained with the lipidic cubic phase method reveal a typical molecule packing of type I, consisting of a well-ordered stacking of 2D-crystals **(a.)** The 2D-crystals contact each other through the glycosyl groups of Asn2 and Asn15 of the rhodopsin N-termini, generating head-to-head crystal contacts in the c-dimension. **(b.)** View of the potential physiological dimer^35^ contacting the transmembrane domains 1 (in blue) of each of the two rhodopsin molecules. **(c.)** top view of the molecules arrangement. The “Spectrum” rainbow colour transforms gradually from the TM1 in blue to the TM7 and the amphipathic helix 8 in red. **d-i)** Lattice translation correction. Despite a straightforward molecular replacement (Phaser MR, Phenix^75^, see Methods) and a solution harbouring a P 2 2_1_ 2_1_ space group, the dark rhodopsin data analysis indicated the presence of more than one off-origin peaks in the Patterson function^36^. In particular, the Patterson peak at td = (0,0.245,0)(SwissFEL) and (0,0.243,0)(SACLA) were attributed to the presence of two translation-related domains within the crystals. Accordingly, a ghost density was identified (**d**) in the Fo-Fc map (in green and red, contoured at 3.83 rmsd) partially overlapping with the 2Fo-Fc map (blue, 2.7 rmsd) from which the rhodopsin model was built in. **e)** After correction of the single domain X-ray intensities^36^ according to Wang et al.^72^ (see Methods), the overall rhodopsin electron density map 2Fo-Fc displays less ghost electron density (green and red). More locally at a few affected amino acid residues locations, e.g. compare the electron density of P34^(1.29)^/A in the lower panel **(g)** (corrected) to upper **(f)** (original). **h-i)** Importantly, the retinal binding pocket of rhodopsin was not affected by the density overlapping and correction, and the density after correction **(i)** shows only minor changes in the difference map compared to the original data **(h)**.

The room-temperature SFX structure of rhodopsin in the inactive dark state was obtained at a resolution of 1.8 Å (Extended Data Table 1 (dark state)) from microcrystals grown in a lipidic cubic phase (LCP). As with most membrane protein structures determined from LCP-grown crystals, these crystals display a type I lattice^34^ forming stacks of protein two-dimensional layers built through hydrophobic interactions (Extended Data Fig. 1a–c). Potentially physiologically relevant dimers of rhodopsin molecules^35^ form contacts between the TM1 and helix 8 (H8) segments of each monomer and are assembled in a head-to-tail manner generating the asymmetric unit. By close inspection of the diffraction data and the resulting electron density maps, the presence of translation-related crystal domains was detected. Measured intensities were corrected to account for this, globally improving the quality and interpretability of the maps^36^ (Methods, Extended Data Fig. 1d–i and Extended Data Table 1). Overall, the SFX structure in the inactive dark state of rhodopsin (Fig. 1a) is very similar to other crystal structures collected at cryogenic temperatures (for example, Protein Data Bank (PDB) 1GZM; root mean square deviation = 0.33 Å on C_α_ atoms)^37^. In contrast to earlier structures solved in cryogenic conditions, the present room temperature structure reveals electron density for all of the previously described functional and structural water molecules. These include the water-mediated cluster around the ancestral counterion Glu181 and its polar tyrosine cage (Fig. 1b), which have a role later in the photoactivation process^38,39^. Moreover, a new ordered water molecule was resolved near the Schiff base connecting the proximal counterion Glu113^3.28^ to Met86^2.53^ (Fig. 1c) and Ala117^3.32^. This interaction has a central position at the TM2–TM3–TM7 interface in the TM bundle.

## A picosecond light-induced bent retinal

**Fig. 2:**
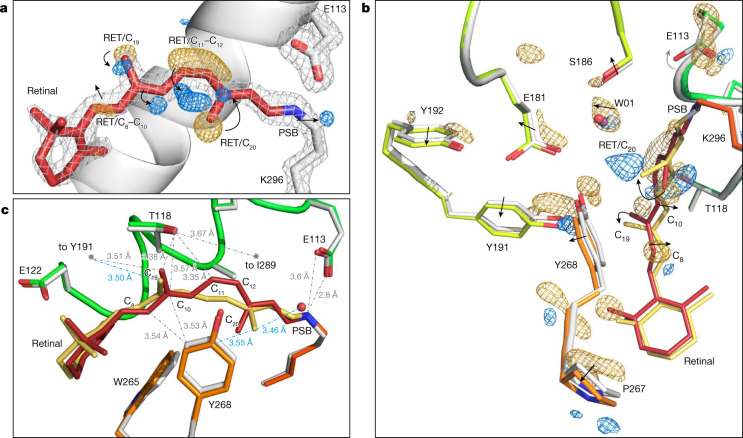
Retinal conformation captured 1 ps after rhodopsin photoactivation using TR-SFX. **a**, Changes in electron density. The retinal (RET) model (red) and the contoured grey mesh (at 2.7*σ* of the 2*F*_obs_ − *F*_calc_ electron density map) correspond to rhodopsin in the dark state obtained by SFX. The difference Fourier electron density (*F*_obs_(light) − *F*_obs_(dark) contoured at 3.8*σ*) around the C_11_=C_12_ bond of the polyene chain and the C_20_ methyl show features appearing after 1 ps photoactivation in blue (positive density) that are correlated with disappearing features in gold (negative density), establishing that the chromophore has already isomerized. A negative density is also observed along C_8_ and C_10_ of the retinal polyene chain. **b**,**c**, The effect of retinal isomerization on the surrounding amino acid residues. The model of 1-ps-photoactivated rhodopsin (retinal in yellow; rhodopsin in orange and green) obtained from the extrapolated map 2*F*_ext_ − *F*_calc_ (21% photoactivation; Methods) superimposed to the dark-state model (retinal in red; rhodopsin in grey). The main chain C_ɑ_ atoms of the protein were used for the structural superposition. **b**, The difference electron density map (*F*_obs_(light) − *F*_obs_(dark), contoured at 3.4*σ*) shows the presence of positive and negative electron densities (blue and yellow) around specific amino acids such as Tyr268^6.51^ of the binding pocket. The arrows illustrate shifts or rotations. **c**, The torsion of the retinal polyene chain at C_11_–C_13_ in the direction of Tyr268 (the π-system at the isomerizing bond of retinal (yellow model) is now rotated 90° with respect to that of the dark state^48^ (red model) (Extended Data Fig. 6)) and the bending along C_6_–C_11_. Selected distances from retinal to rhodopsin residues are shown as grey dotted lines for the dark state and as blue dotted lines for the isomerized form.

**Extended Data Fig. 2:**
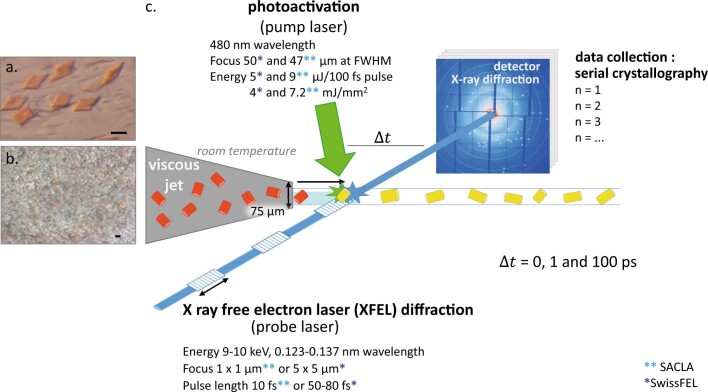
Time resolved serial femtosecond crystallography. Time resolved serial femtosecond crystallography (TR-SFX)^101^ was conducted using an X-ray free electron laser^102^. Crystal plates (20 µm large and about 1.5 µm thick) were made of rhodopsin purified from bovine retinae (**a. panel**, scale bar is 20 µm). The sample was scaled up (**b. panel**, scale bar is 20 µm) and subjected to a pump and probe experiment (**c. panel**) triggered by a photoactivation at 480 nm (pump laser). Briefly, rhodopsin crystals grown in lipidic cubic phase (LCP) in the darkness were successively injected (viscous jet) in the light of a pump laser and probed for X-ray diffraction after various time-delays (∆*t* from 1 to 100 picoseconds) using the X-ray free electron laser from SACLA (Japan)(blue double asterisk) or SwissFEL (Switzerland)(purple asterisk) under the regime “diffract-before-destroy”^103^. The 10 ps time-delay was performed at the SwissFEL with the following laser settings: pump laser, 483 nm, 3 µJ/100fs pulse, 84 µm FWHM and an XFEL at 12 keV with 25 fs pulse length, focus 3 x 5 µm. The processing was done both on the fly and at home using the CrystFEL software^69^ combining 30’000 of the diffraction patterns generated by each crystal into a dataset. The inset of the **b. panel** illustrates the opacity of the lipidic phase around the crystal when the rhodopsin crystals are produced in large quantity, hampering spectroscopic experiments on the TR-SFX sample.

**Extended Data Fig. 3:**
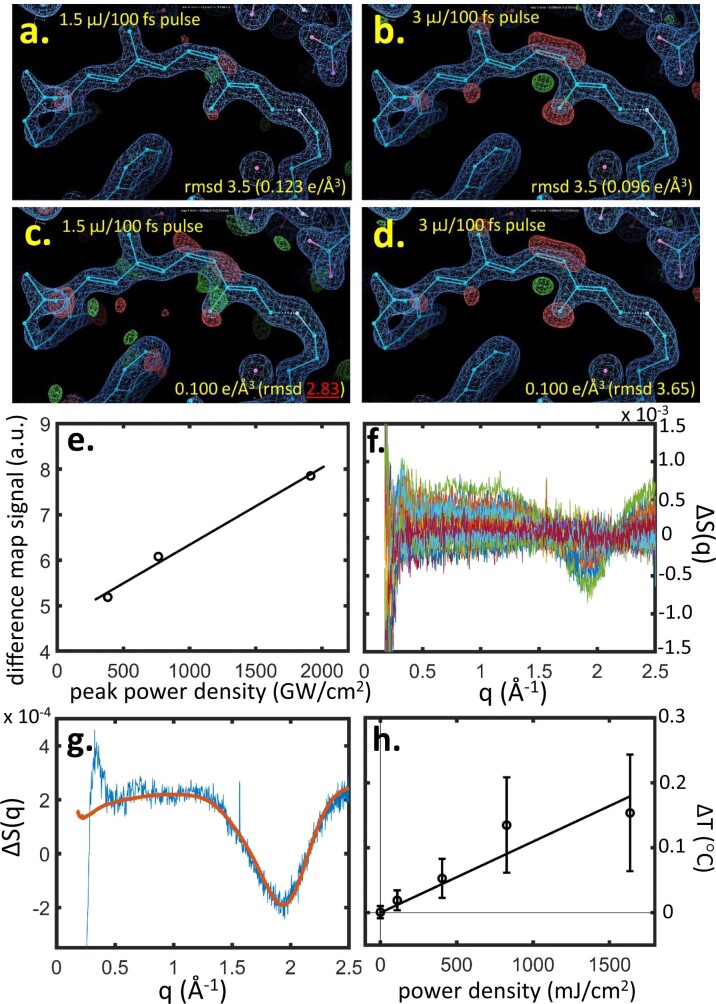
Correlation between the power density of the pump laser and either the water heating signal by time-resolved X-ray solution scattering (TR-XSS) or the rhodopsin photoproduct appearance by time-resolved serial femtosecond crystallography. **a-e.)** Lowest limit of pump laser power (a-e) for observing decent difference electron density (DED) signals in rhodopsin within the present experimental settings. Two datasets of rhodopsin photoactivated for a 10 ps time delay were collected (SwissFEL beamtime 20200597) at 2 different laser powers, 1.5 (**a and c**) and 3 µJ/100 fs pulse (**b and d**) with the same focus (100 µm spot diameter (1/e^2^)) and same amount of 29.900 images. For both pump laser powers, the retinal isomerization and the concomitant C20-methyl rotation are observable, but the positive electron density signals are barely detectable in the case of the low power setting (**a panel compared to b**), e.g. the 11-cis-to-trans event is marked by a strong negative electron density on the 11-cis, but no corresponding positive density is detected. When the intensity of the DED is increased for both until an equal level of 0.100 e/Å^3^, we observe that the signal-to-noise ratio for the low pump laser energy condition (**c panel**) is so low that the signals cannot be interpreted, compared to the condition with twice the energy (**d panel**). Power titration including 1.5 µJ/100 fs pulse, 3 µJ/100 fs pulse (10 ps time points) and the 5 µJ/100 fs pulse (1 ps time point), with peak power density of 382 GW/cm^2^, 764 GW/cm^2^ and 1914 GW/cm^2^, respectively (**e panel**). **(f-h)** Time-resolved X-ray solution scattering (TR-XSS) studies of visual rhodopsin using XFEL radiation. **f)** TR-XSS difference data (laser on minus laser off) recorded at the LCLS from detergent solubilized samples of rhodopsin for the time-delays 10 ps ≤ Δt ≤ 1 μs at various laser power densities. **g)** Principal singular value decomposition (SVD) component (blue line) from samples of visual rhodopsin indicating laser induced heating (characteristic curve from 0.5 Å^−1^ ≤ q ≤ 2.5 Å^−1^) as well as oscillations usually associated with protein induced structural changes (visible from 0.25 Å^−1^ ≤ q ≤ 1.0 Å^−1^). An experimental difference X-ray scattering curve due to heating alone (red line) recorded from detergent solubilized samples of a photosynthetic reaction centre using synchrotron radiation^65^, is used to calibrate this laser induced heating. **h**) Laser induced heating in samples of visual rhodopsin measured by TR-XSS when using a 480 nm fs laser pulse with a fluence of 110 mJ/cm^2^ (number of independent measurements, n = 6); 400 mJ/cm^2^ (n = 6); 830 mJ/cm^2^ (n = 3); and 1640 mJ/cm^2^ (n = 2), where the fluence is averaged across the FWHM of the laser spot. Negative time-delays (n = 4) were used as a control (plotted at zero fluence). Data are presented as mean values +/− SEM.

**Extended Data Fig. 4:**
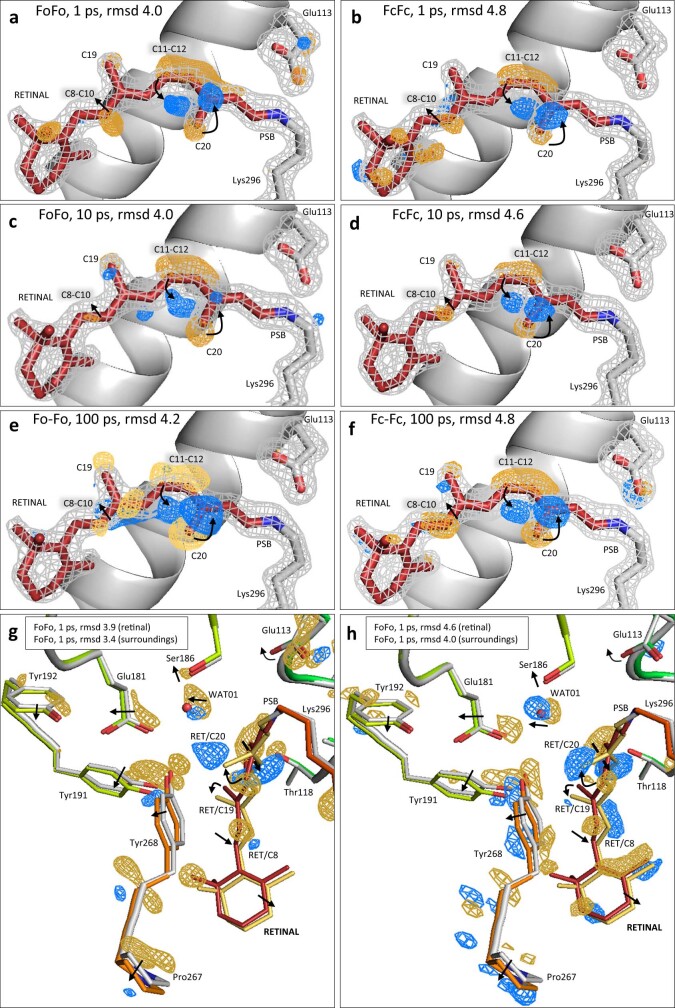
Comparison of observed and calculated difference electron density maps at the three time-delays 1, 10 and 100 ps of rhodopsin photoactivation, showing that the refined models (right panels) are in good agreement with raw difference electron density data (left panels). Difference Fourier electron density maps were created directly from the starting experimental electron density maps (Fobs(light)-Fobs(dark)) **(left panels)** or from the calculated atomic structure model factors (Fcalc(light)-Fcalc(dark)) **(right panels)** and compared for the 1 ps dataset **(a versus b; g versus h)**, 10 ps **(c versus d)**, 100 ps datasets **(e versus f)**. The panels show the retinal binding pocket of rhodopsin in the dark state (**all panels**) (or superimposed with the 1 ps photoactivated structure **(g-f)**) with retinal in red (or in yellow for the 1 ps structure **(g-f)**) and contoured with the 2Fobs-Fcalc electron density map (grey mesh) **(a—f)**. Highlighted in colour, the difference Fourier electron density signals between photoactivated and dark rhodopsin are displaying features appearing with time, in blue (positive density) that are correlated with disappearing features in gold (negative density). The mesh contouring at various rmsd values was adjusted for easier side to side comparison of the different types of maps and clarity of the figure (see the values in the panels **a—h**).

**Extended Data Fig. 5:**
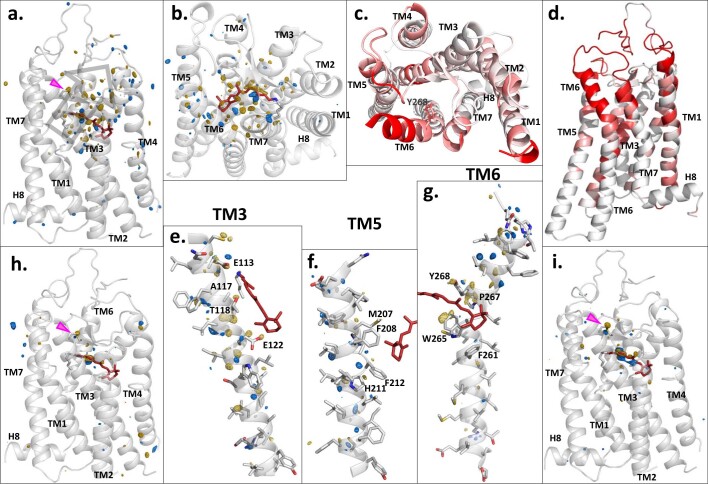
Anisotropic breathing motion of rhodopsin. Comparison of the overall conformational changes in rhodopsin photoactivated for 1, 10 and 100 picoseconds. The difference electron density map (Fobs(1ps-light)-Fobs(dark) contoured at 4.2 rmsd) from the dataset of 1ps-illuminated rhodopsin superimposed on the rhodopsin dark state structure model (grey) (**a-b**) shows strong signals (blue=positive density; yellow=negative density) on the retinal molecule (red) demonstrating the early isomerization. Surrounding the retinal, changes occur at the amino acid level in an anisotropic direction towards the extracellular side (grey arrow of **panel a**) along TM5 and TM6 (**panels f and g**). This anisotropic breathing motion can be detected in the extracellular part of TM3 (**e**), TM5 (**f**) and TM6 (**g**). After 10 ps (**panel h**) and 100 ps (**panel i**) of photoactivation, most of the conformational concerted motion changes are reset (**h, i and** Extended Data Table 3), only a few amino acids only will not revert –like the disulphide bridge C110-C187 (pink arrow)- and take part to further changes along the photoactivation pathway (Extended Data Table 3). Interestingly, we also observe localized intrinsic fluctuations in molecular dynamics simulations of rhodopsin in the dark state (PDBid: 1GZM) analysed from the GPCRmd database^98^. These fluctuations localize at the extracellular side of the transmembrane bundle and are compatible with the energy dissipation changes observed at 1 ps (compare **panels c and d (molecular dynamics simulations) with the panels a and b (breathing motion)**). Three independent molecular dynamics simulations (3 x 2500 frames) of rhodopsin with retinal (PDBid: 1GZM) were analysed and the backbone root-mean-square fluctuation (RMSF) was depicted on each residue with increasing values from white to red (the RMSF scale was truncated at 1.8 Å for clarity).

The first metastable intermediate of rhodopsin (bathorhodopsin, Batho-Rh)^2,15,40^ arises 200 fs after photoactivation. It is fully populated by ∆*t* = 1 ps (refs. ^14,41^) and persists for tens of nanoseconds^42,43^. To characterize the structure of Batho-Rh, we collected TR-SFX data at the Swiss and Japanese XFELs (Extended Data Fig. 2) from LCP-grown microcrystals of rhodopsin photoactivated under a regime in which laser-induced heating is low (Extended Data Fig. 3, Methods) and in which we recovered high-quality difference electron density maps below the maximum activation (Extended Data Fig. 3) using a femtosecond-pump laser with a 480-nm wavelength for three time delays of photoactivation of ∆*t* = 1 ps, 10 ps and 100 ps. High-quality TR-SFX data (Extended Data Table 1) are represented as difference Fourier electron density maps in Fig. 2a,b and Extended Data Fig. 4. For the shorter time delay, changes in electron density are distributed in a highly anisotropic manner, clustering in the immediate vicinity of the buried retinal chromophore and propagating towards the cytoplasmic side of the protein through the TM5 and TM6 helices (Extended Data Fig. 5) at a minimum speed of 18 Å ps^−1^ measured along TM6 (1,800 m s^−1^, slightly above the speed of sound in water and in accordance with the speed of sound in ribonuclease A crystals^44^). This structural anisotropy had completely decayed by Δ*t* = 100 ps.

**Extended Data Fig. 6:**
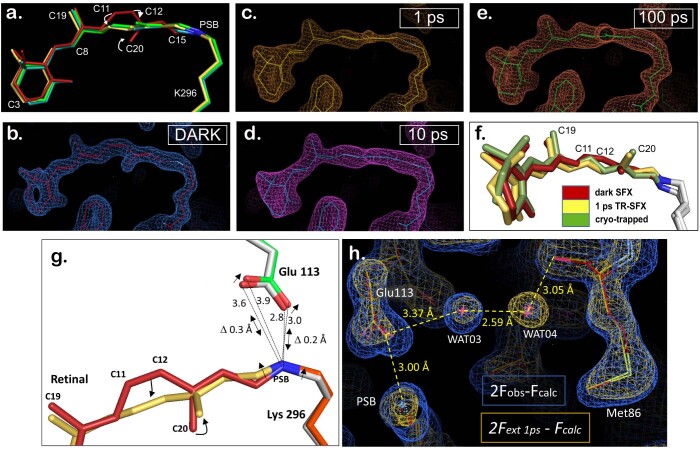
Conformation of retinal after 1, 10 and 100 ps of rhodopsin photoactivation using TR-SFX, and Schiff base surroundings. **(a-f)** Retinal conformational changes until 100 ps. **a)** The superimposition of the retinal TR-SFX models in the dark (red model) and 1 to 100 ps photoactivation time delays highlights the main differences: the cis-to-trans isomerization at C11-C12 and the concomitant rotation of the C20-methyl around C13. Beside a slight tilt of the β-ionone ring, another difference between 1 (yellow model), 10 (light blue) and 100 (green) ps-structures is a slight relaxation of the polyene chain towards planarity. **b)** Original electron density map around the retinal in the rhodopsin dark state obtained by SFX (2Fo-Fc map contoured at 2.5 rmsd) and the resulting refined model in red. **c)** Extrapolated electron density map around the retinal of 1 ps-photoactivated rhodopsin obtained by TR-SFX (2Fext-Fc map contoured at 1.9 rmsd) and the resulting refined model in yellow. **d)** Extrapolated electron density map around the retinal of 10ps-photoactivated rhodopsin obtained by TR-SFX (2Fext-Fc map contoured at 0.9 rmsd) and the resulting refined model in blue. **e)** Extrapolated electron density map around the retinal of 100 ps-photoactivated rhodopsin obtained by TR-SFX (2Fext-Fc map contoured at 1.9 rmsd) and the resulting refined model in green. **f)** Structure of rhodopsin after 1ps photoactivation (yellow model) obtained by TR-SFX (this study) compared to a cryo-trapped bathorhodopsin state (green model) and the dark state (red model). **(g-h)** Schiff base-counterion E113 and neighbouring water hydrogen bond network after 1 picosecond of photoactivation. **Panel g**: influence of the C11-C12 isomerization on the Schiff base conformation and distance to the counterion E113. The two models of rhodopsin are superimposed on the Cɑ atoms of the protein. Retinal after 1 ps of photoactivation (yellow (with orange K296^(7.43)^)) is showing an all-trans conformation and the C15 of the C14-C15-NZ plane at the SB displays a slight kick towards the extracellular space compared to the structure of the dark state (red). The counterion E113^(3.28)^ moves accordingly, in the same direction of about 0.2-0.3 Å. **Panel h**: of the two water molecules W03 and W04 which form a bridge between the counterion E113^(3.28)^ and M86^(2.53)^ (and also contacting A117^(3.32)^, F91^(2.58)^ and F116^(3.31)^, not shown), only W04 has gained order. The two rhodopsin molecules models (dark in red; 1 ps in yellow) are contoured with their respective electron density maps, in blue (2Fo-Fc contoured at 1.3 rmsd) and in orange (2Fextrapolated-Fc contoured at 1.3 rmsd). By Δ*t* = 100 ps we observe a reset of the occupancy, which is similar to that of the dark state structure.

Light-induced structural changes within the retinal polyene chain are observed at ∆*t* = 1 ps as a strong negative difference electron density feature (minimum of −6.2*σ*, where *σ* is the root mean square electron density of the unit cell) and a complementary positive difference electron density feature (maximum of +5.8*σ*) (Fig. 2a) associated with the C_11_=C_12_ double bond revealing that this bond has isomerized. This event is associated with changes in electron density near the C_20_ methyl (−6.6*σ* and +5.6*σ*). Modelling these electron density changes in combination with structural refinement against crystallography observations extrapolated to 100% occupancy of the photoactivated intermediate (for photoactivation levels, see the Methods and Extended Data Table 1; 1-, 10- and 100-ps time delays) establishes that retinal isomerization involves a large (47.7°) clockwise rotation (as viewed from the PSB) of the C_20_ methyl (or anticlockwise if adopting the conventional dihedral angle sign with view from the β-ionone side). This rotation happens towards the extracellular side in concert with a shift in both the proximal water molecule W01 and Tyr268^6.51^ of the Glu181 cage (Fig. 2b). The tilt of the C_20_ methyl increases (Extended Data Fig. 6e) to 51.3° by ∆*t* = 100 ps. A similar distortion was measured as 54.9° in a cryo-trapped Batho-Rh state study that lacked temporal resolution^10^ (Extended Data Fig. 6f). We conclude that, under the used illumination conditions, a stable structural state is generated. Although direct evidence that excludes the influence of multiphoton absorption on the observed structural states cannot be readily obtained, it suggests that the observed structural changes are qualitatively correct. The plane of retinal containing the C_19_ methyl, which is located on the opposite side of the isomerizing C_11_=C_12_ bond, is fixed in the resting state by Thr118^3.33^ (3.36 Å away)^45,46^ and Try191^ECL2^. Consequently, the C_19_ methyl is only minimally affected by the *cis*-to-*trans* isomerization (Fig. 2 and Extended Data Fig. 6), shifting only half an angstrom towards Tyr191^ECL2^ (with a 36.0° rotation compensated by a backwards elbow movement of the polyene chain) and in the opposite direction to the C_20_ methyl (Fig. 2b,c).

These rearrangements in the retinal molecule at Δ*t* = 1 ps are compatible with an aborted ‘bicycle pedal’ mechanism of photoisomerization^14,47–50^ in which the interactions of the C_19_ methyl of retinal with specific residues in the tight binding pocket confer resistance to a larger rotation of this methyl group. The consequence for such aborted C_9_=C_10_ isomerization is the release of energy over the polyene chain, affecting mostly C_8_ and C_10_, which elbow in the opposite directions relative to the C_20_ methyl (Fig. 2b,c). The C_6_–C_11_ segment of the retinal polyene chain is bent, aligning all carbons in a near-perfect arc (Fig. 2c), affecting the surrounding interactions in the binding pocket.

The absorption maximum of this state was computed by quantum mechanics/molecular mechanics (QM/MM) optimization of the experimental structure at Δ*t* = 1 ps, and a subsequent vertical excitation energy calculation between the ground and the optically active first excited state of the PSB (Methods). This yielded a spectral red-shift of 32 nm relative to the dark state (for an extended QM system composed of the retinal PSB, Glu113^3.28^, Glu181^ECL2^, Tyr191^ECL2^, Tyr268^6.51^, Ser186^ECL2^ and water W01) that is in good agreement with the experimental red-shift of 31 nm (ref. ^51^) for Batho-Rh (Extended Data Table 2). Notably, one of the first QM/MM calculations on bovine rhodopsin using the CASPT2//CASSCF/AMBER method on a simple QM system (retinal PSB) yielded a red-shift of about 22 nm (ref. ^48^), which is comparable to our calculated value of 24 nm in an equivalent QM system. Our calculations on the extended QM system also show that the twisted all-*trans* retinal at 1 ps stores an excess of 36 kcal mol^−1^ of energy compared with the planar 11-*cis* conformation (for comparison, the energy of a 480-nm photon is 59.6 kcal mol^−1^), which is in good agreement with the experimental value of 32 kcal mol^−1^ measured for the rhodopsin-to-Batho-Rh transition^52^.

## Rhodopsin binding pocket at 1 ps

**Fig. 3:**
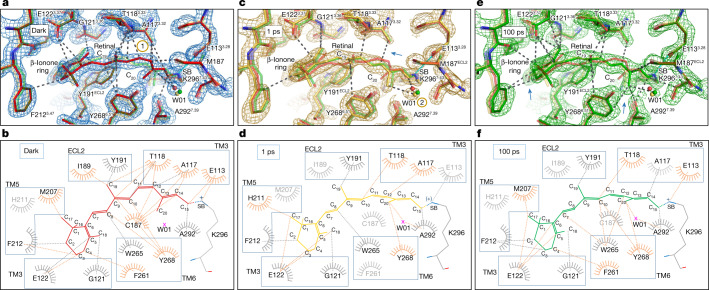
Residue environment distances from retinal measured using PyMol and LigPlot software, respectively. **a**,**c**,**e**, The two rhodopsin models after 1 ps (yellow) and 100 ps (green) of photoactivation were superimposed in PyMol with the rhodopsin dark-state model (red) and the residue environment distances were drawn with a cut-off at 3.7 Å using dashed lines from the retinal in the dark (**a**) and after 1 ps (**c**) and 100 ps (**e**) photoactivation. The salt bridge between the Schiff base (SB) and counterion Glu113 is marked in cyan. The yellow-circled numbers 1 and 2 show the regions in which interactions will weaken and appear, respectively (see also the blue arrows). **b**,**d**,**f**, While PyMol displays the three-dimensional structure of amino acids in the previous panels, the LigPlot^58^ represents a flat interaction plot with all amino acids involved (orange) or less (grey) in the conformational changes during the picosecond time delays of photoactivation (dark (**b**), 1 ps (**d**) and 100 ps (**f**)).

**Extended Data Fig. 7:**
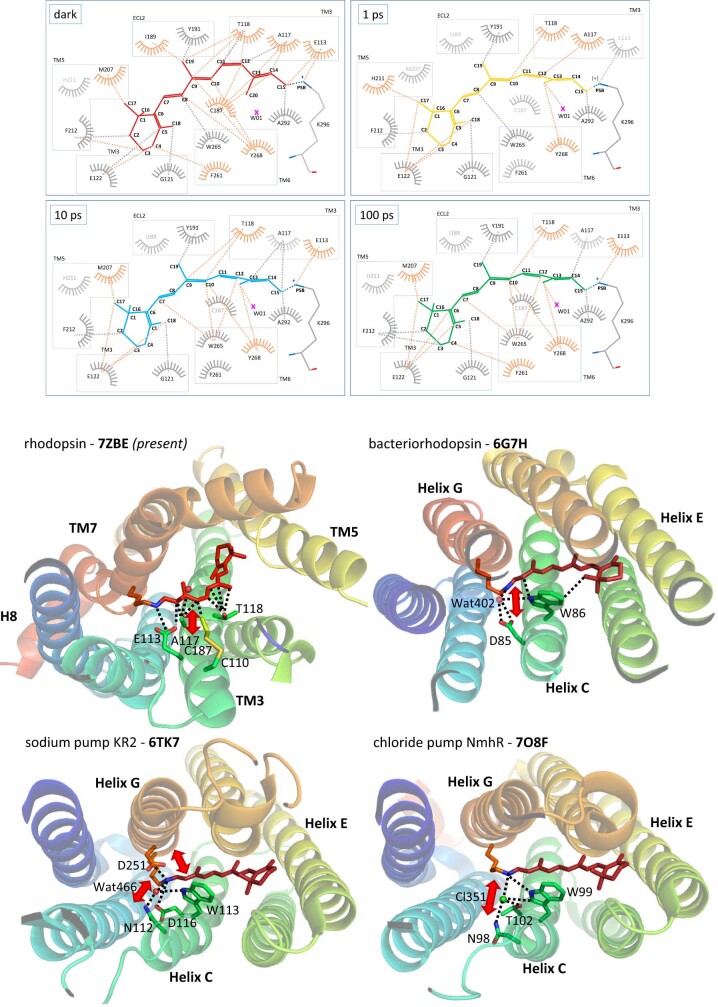
Interactions of retinal with its binding pocket at 0, 1, 10 and 100 ps of photoactivation and comparison with microbial rhodopsins. LIGPLOT^58^ view of retinal interactions within the rhodopsin binding site **(upper four panels)** (set distance < 3.6 Å) at different time-delays of photoactivation, dark state, 1, 10 and 100 ps-photoactivated states. The amino acids and dashed lines of interatomic interactions labelled in orange are the site of major changes, showing new interactions, e.g. with water W01 or losing contact with C187^(ECL2)^. Retinal binding pocket of non-homologous rhodopsins **(lower four panels)** (PDBid codes shown in bold) from mammalian (the rhodopsin GPCR from Bos taurus (this study) (PDBid=**7ZBE**)) and prokaryotes (bacteriorhodopsin proton pump from Halobacterium salinarum (PDBid=**6G7H**); KR2 sodium pump from *Krokinobacter eikastus* (PDBid=**6TK7**) and the NmHR chloride pump from *Nonlabens marinus* (PDBid=**7O8F**)). The third transmembrane helix (“TM3” in GPCRs and “helix C” in prokaryotes) has been described as a main interaction site for retinal^104^, often carrying the stabilizing counterion, like E113 in rhodopsin. Projected on the dark state structure of these four rhodopsins, some important structural rearrangements upon retinal isomerization (*cis*-to-*trans* for mammalian rhodopsin, *trans*-to-*cis* for prokaryotic rhodopsins), observed in TR-SFX studies in the picosecond range, are indicated with a red arrow. The disruption of these important interactions weakens the stabilization of the retinal chromophore by TM3/helix C.

Light-induced isomerization transforms retinal into an agonist that interacts differently with residues in the rhodopsin binding site. One picosecond after photoactivation, the isomerized all-*trans* retinal fills the same volume as the 11-*cis* resting conformation, confirming the hypothesis of space-saving motion^47^, but is now free from several hydrogen bonds and van der Waals interactions that stabilize the dark-state structure in an inactive conformation (Fig. 3a,b). The covalently bound retinal bends like an arc stabilized in the middle by steric hindrance and van der Waals interactions between the C_19_ and C_20_ methyl groups of the retinal and two tyrosine residues from the ancestral counterion network, Tyr191^ECL2^ and Tyr268^6.51^. The isomerization and rotation of the C_20_-methyl–C_13_–C_14_ plane induce a kink of C_15_ from the polyene chain that influences only minimally the neighbouring PSB/Glu113^3.28^ salt bridge, except the order of the surrounding water W04 in the Glu113 hydrogen bond network (Fig. 2c and Extended Data Fig. 6g,h). The interactions between the polyene chain and Ala117^3.32^–Thr118^3.33^ are two critical retinal contacts with TM3 that are weakened at Δ*t* = 1 ps (compare Fig. 3a,b with Fig. 3c,d; Extended Data Fig. 7 (top) and Supplementary Video 1). An additional interaction with TM3 is disrupted, between the isomerizing bond and Cys187^ECL2^ of the structurally important and highly conserved disulfide bridge Cys187–Cys110 (ref. ^53^), linking TM3 to ECL2 (compare Fig. 3a,b with Fig. 3c,d). Notably, the light-induced structural changes observed around the retinal of bovine rhodopsin bear some topological resemblance to those observed in other low-homology seven-TM-helix retinal-binding proteins from bacteria and archaea. For example, a weakening or disruption of some interactions between retinal and TM3 (which corresponds to helix C in prokaryotic opsins) is also observed (Extended Data Fig. 7 (bottom)) in bacteriorhodopsin^24^, the sodium photosensitive pump KR2^24,26^ and the chloride pump *Nm*HR^27^, which all undergo completely different activation mechanisms. Thus, despite their different evolutionary origins and types of isomerization (11-*cis*-to-all-*trans* versus all-*trans*-to-13-*cis*), retinal-binding proteins seem to share the need to disengage the retinal from the central TM helix before undergoing the next steps of activation.

**Fig. 4:**
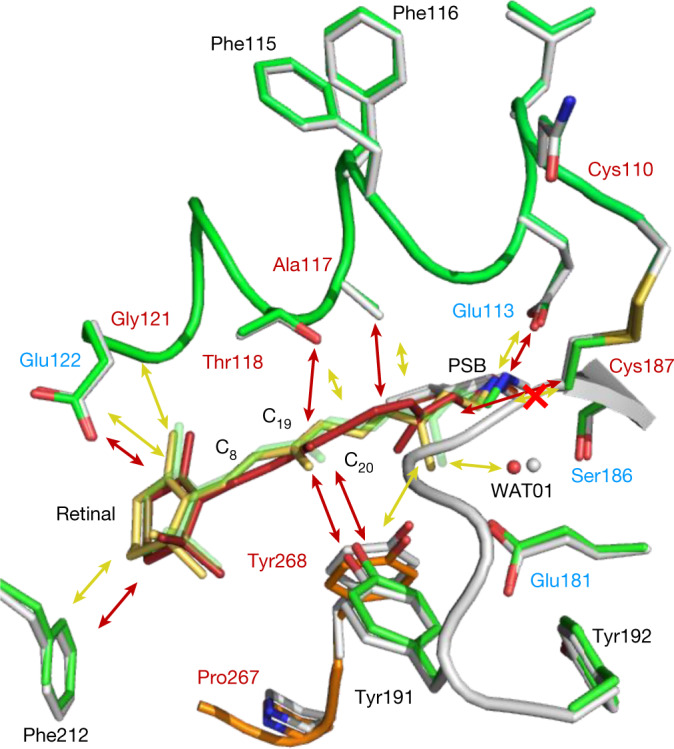
Interactions between retinal and the binding-pocket residues are substantially reduced 1 ps after photoactivation. Schematic of the interactions of retinal in the rhodopsin ligand-binding pocket before (red arrows) and after photoactivation in the picosecond range (yellow arrows; the red cross on the yellow arrow shows the bond disruption). A longer arrow represents a stronger interaction. The grey structure corresponds to the dark state (retinal in red) and the coloured structure corresponds to the 1-ps illuminated model (retinal in yellow). For comparison, the retinal model after 100 ps is shown in green. The residues labelled in red are GPCR-conserved and the blue residues are rhodopsin-conserved.

As mammalian rhodopsin evolves along its reaction pathway, some of the observed changes will become more pronounced while others will revert (Fig. 4 and Extended Data Table 3). By Δ*t* = 10 and 100 ps, the β-ionone ring and C_19_–C_20_ methyl groups still interact respectively with Gly121^3.36^–Glu122^3.37^ and the tyrosines of the Glu181^ECL2^ polar cage (Fig. 3e,f and Extended Data Fig. 7 (top)), while, for example, Tyr268^6.51^ and Pro267^6.50^ of TM6 relax to their initial positions. Notably, the observed anisotropic character of energy dissipation through TM6 is compatible with intrinsic structural fluctuations in molecular dynamics simulations of rhodopsin in the dark state (Extended Data Fig. 5c,d). This helix is relatively rigid towards the intracellular G protein-binding site but noticeably more flexible towards the extracellular domains, with the key Pro267^6.50^ at the joint.

Whereas rhodopsin is considered to be a prototypical class A GPCR, the mechanism used by GPCRs to recognize diffusible agonist ligands by conformational selection is vastly different from the extreme case of induced fit displayed by light-activated GPCRs such as rhodopsin^54^. Notably, both conformational selection and induced fit converge rapidly into a common GPCR-activation mechanism^55^ and the early stages of retinal isomerization may therefore reveal fundamental determinants of agonism in GPCRs. For example, early structural changes in the retinal-binding pocket are associated with a small outward tilt (about 0.5 Å) near Pro215^5.50^ and Pro267^6.50^, located around the middle of TM5 and TM6 (Fig. 2b) and enable anisotropic motions in the extracellular part of the receptor (Extended Data Fig. 5). Both proline residues are conserved in class A GPCRs and are key in agonist-induced activation^56^. Moreover, TM3 is a region that has a central role in the architecture of the TM bundle of class A GPCRs^57^ by forming part of both the ligand and the G protein-binding pockets. Our TR-SFX data reveal that, even at an early stage of activation, the inverse agonist (11-*cis* retinal) has stripped itself away from TM3 (Figs. 3 and 4 and Supplementary Video 1) while isomerizing into the agonist conformation (Fig. 3c,d). Several of the affected positions in TM3 (Glu113^3.28^, Thr118^3.33^, Gly121^3.36^ and Glu122^3.37^) correspond to conserved residues in the binding site of class A GPCRs that are involved in ligand binding^57^, with Gly121^3.36^ in particular being part of a consensus ‘cradle’ scaffold for ligand recognition^57^. Thus, our TR-SFX observations using XFEL reveal how the photoactivated conformation of the retinal rapidly weakens many van der Waals interactions with the amino acids of the rhodopsin binding pocket (Fig. 4) and thereby commits the receptor’s relaxation pathway towards its G protein-binding signalling conformation.

## Conclusions

Our high-resolution SFX structure of rhodopsin in the inactive dark state at room temperature reveals the entirety of the water-mediated hydrogen bond network within the protein. One picosecond after light activation, rhodopsin has reached the red-shifted Batho-Rh intermediate. Already by this early stage of activation, the twisted retinal is freed from many of its interactions with the binding pocket while structural perturbations radiate away as a transient anisotropic breathing motion that is almost entirely decayed by 100 ps. Other subtle and transient structural rearrangements within the protein arise in important regions for GPCR activation and bear similarities to those observed by TR-SFX during photoactivation of seven-TM helix retinal-binding proteins from bacteria and archaea. We therefore suggest that the protein disperses an initial excess of energy through the early GPCR structural pathways that will be used for activation. Our study reveals an ultrafast energy dissipation in rhodopsin occurring through conserved residues of GPCR activation pathways and lays the experimental groundwork to study the early activation events in the large family of class A GPCRs.

## Methods

### Rhodopsin extraction from retinae and purification

All extraction and purification steps were carried out under dim red light conditions. Commercially available dark-adapted frozen bovine retinae (W L Lawson Company) were used to isolate rod outer segment (ROS) membranes according to a protocol described previously^59^. In brief, 200 frozen bovine retinae were diluted in ROS buffer (10 mM MOPS, 30 mM NaCl, 60 mM KCl, 2 mM MgCl_2_, 1 mM DTT), 40% (w/w) sucrose and two tablets of cOmplete protease inhibitor cocktail. The mixture was shaken by hand for 4 min, centrifuged at 4 °C, 4,000*g* for 30 min. This step was repeated and the pooled supernatants were combined, diluted by a half with ROS buffer containing no sucrose and centrifuged at 4 °C, 24,000*g* for 30 min. The pellets were resuspended in ROS buffer containing 23.4% sucrose and layered onto a freshly prepared gradient with two layers of ROS buffer with 34% (w/w) and 29% (w/w) sucrose. The ROS-membrane-loaded gradients were centrifuged using a swing-out rotor SW28 at 4 °C at 110,000*g* for 90 min and rhodopsin-containing layers (23–29% interface and 29% layer) were aspirated and flash-frozen in liquid nitrogen. The rhodopsin concentration of the dark-state ROS membranes, determined by recording a UV/VIS spectrum before and after illumination, yielded commonly 200 ± 40 mg rhodopsin. Bovine rhodopsin can be further isolated by detergent solubilization and affinity chromatography using a concanavalin A resin (ConA, GE Healthcare Life Sciences) as described previously^60^. This protocol was optimized: (1) the starting retinae material was doubled; (2) the amount of resin was scaled-up three times; (3) to sharpen the elution profile, the second half of the elution phase was performed in reversed flow. A ROS membrane suspension containing around 180 ± 20 mg rhodopsin was diluted three times in ConA buffer (50 mM sodium acetate, 150 mM NaCl, 3 mM MgCl_2_ 6H_2_O, 3 mM MnCl_2_ 4H_2_O, 3 mM CaCl_2_ 2H_2_O, 1 mM Na_2_-EDTA 2H_2_O, 2 mM 2-mercaptoethanol, pH 6) and centrifuged at 4 °C, 104,000*g* for 35 min. The resulting ROS membrane pellet was resuspended in 90 ml ConA buffer containing one tablet of protease inhibitor and the membranes were solubilized at room temperature with lauryldimethylamine-oxide (LDAO, Sigma-Aldrich) to a final concentration of 1%. The solubilized sample was centrifuged at 4 °C, 118,000*g* for 60 min before ConA affinity chromatography in ConA buffer containing 0.1% (w/v) LDAO and direct elution with 0.2 M methyl α-d-mannopyranoside in the same buffer. Aliquots of rhodopsin at 2 mg ml^−1^ were flash-frozen in liquid nitrogen and ready to use for crystallization in LCP.

### Crystallization and TR-SFX sample preparation

All crystallization and sample preparation steps were performed under dim red light conditions. Before crystallization in LCP, flash-frozen aliquots of purified rhodopsin were thawed and the detergent was exchanged for 0.21% *n*-decyl-*N*,*N*-dimethylamine-*N*-oxide (DAO, Anatrace) in 50 mM sodium acetate, 150 mM NaCl, 3 mM MgCl_2_, pH 6.0 using a PD10 buffer exchange column (Sigma-Aldrich). The eluate was centrifuged at 18 °C, 21,000*g* for 15 min and further concentrated to at least 20–25 mg ml^−1^ using a concentrator Ultra 4 MWCO 30 at 18 °C and 4,000*g*. After centrifugation at 18 °C and 21,000*g* for 15 min, the protein sample was mixed at 22 °C with monoolein (1-oleoyl-rac-glycerol, Nu-Check prep) at a ratio of 40 to 60, respectively, using gas-tight Hamilton syringes until formation of the translucid LCP. The well diffracting rhodopsin crystal was initially obtained from a low-molecular-mass polyethylene glycol screen with various types of buffer and the first hit detected using the second harmonic generation imaging SONICC (second order nonlinear optical imaging of chiral crystals) device (Formulatrix)^61^. For crystal growth, 20–40 µl of protein-laden LCP was injected into precipitant-laden Hamilton glass syringes containing 200–400 µl of precipitant (37–39% PEG 600 and 100 mM Bicine pH 9.0). Alternatively, 80 µl of protein-laden LCP were injected into 1 ml plastic syringes containing 800 µl of precipitant. The samples were wrapped in aluminium and stored at 18 °C. After 3 days, plate-shaped crystals in LCP with dimensions of about 15 × 15 × 1.5 µm can be collected by removing the precipitant and kept stable for weeks in the darkness at 18 °C.

At the X-ray free-electron laser beamline, the LCP sample containing rhodopsin crystals was imperatively mixed with a three-way coupler to ensure homogeneity^62^ before loading into a reservoir of the high-viscosity injector. In case of residual precipitant contamination, the LCP-laden crystal sample was titrated with PEG 1,000 (50% (w/v)) and finally mixed 1:5 with monoolein.

### Time-resolved pump probe serial crystallography

X-ray diffraction data were collected at the XFELs BL3_EH2 end station of the SACLA^63^ (beamtimes 2015B8043 and 2018A8066) and Alvra end station of the SwissFEL (beamtimes 20172060 and 20200597). The energy of the X-ray beam was 9–10 keV with a pulse length of 10 fs (SACLA) and 65 fs (SwissFEL) and a focus at the sample of 1 × 1 µm (SACLA) and 5 × 5 µm (SwissFEL). The hutch was prepared for dim red-light conditions and the femtosecond pump laser set at a wavelength of 480 nm with a pulse energy of 9 (SACLA) to 5 (SwissFEL) µJ per 100 fs pulse duration at the sample position. The pump laser beam size was of 47–50 µm FWHM (80–85 µm 1/e^2^). If one assumes idealized Gaussian beam optics, then this corresponds to a peak energy density of 200 mJ cm^−2^ at SwissFEL (Δ*t* = 1 ps) or, alternatively, a mean energy density of 140 mJ cm^−2^ averaged over the laser’s focal FWHM (compare with tabulated data in ref. ^3^; or a peak power density of 2,000 GW cm^−2^). The corresponding values for the SACLA study (Δ*t* = 100 ps) are a peak fluence of 360 mJ cm^−2^, and average fluence of 260 mJ cm^−2^, and a peak power density of 3,600 GW cm^−2^. The experimental illumination conditions were chosen to generate a high occupancy of these observed structural states that evolve over time. Although these conditions would be deemed to be excessive in spectroscopic experiments, if one calculates the product of the average fluence (*F*) with the resting state absorption cross-section (*σ*) divided by the energy of a single photon (*hν*, where *h* is Planck’s constant and *ν* the photon’s frequency) we recover *σF*/*hν* = 45 for the illumination conditions at SwissFEL and *σF*/*hν* = 81 for those used at SACLA. Whereas these values may suggest considerable multiphoton excitation, the results from time-resolved X-ray solution scattering studies on rhodopsin (Extended Data Fig. 3) imply that fewer absorbed photons lead to heating in the sample (see the ‘Time-resolved X-ray solution scattering’ section below). Moreover, the use of 60% of the energy of the pump laser (3 µJ per 100-fs pulse, 764 GW cm^−2^) in the 10-ps time delay indicated the lowest energy applicable in our TR-SFX study (Extended Data Fig. 3) and a photoactivation regime higher than single photon per rhodopsin, leaving open the possibility that nonlinear effects contributed to the observed photochemistry.

Crystals of bovine rhodopsin grown in LCP were used to collect TR-SFX data at time delays of 1 ps, 10 ps (SwissFEL) and 100 ps (SACLA) (Extended Data Fig. 2). The crystals were extruded using a high-viscosity injector through a 75-μm nozzle with a constant flow rate of 0.033 μl min^−1^ (SwissFEL) or 2.5 μl min^−1^ (SACLA)^64^ to the pump probe intersection point where the data were collected with every fifth shot of the pump laser blocked (data collection scheme of 4 light-activated, then 1 dark) (SwissFEL) or interleaving ON/OFF-laser (collection in the mode 1 light:1 dark (SACLA)), depending on the repetition rates of the XFELs (SwissFEL 25 Hz; SACLA 30 Hz) and pump lasers (SwissFEL 25 Hz; SACLA 15 Hz), respectively. As a control, true dark-state data were also collected with the pump laser off (all dark data, SFX mode).

### TR-XSS analysis

TR-XSS studies using samples of detergent-solubilized rhodopsin injected using a gas dynamics virtual nozzle (GDVN) liquid microjet were performed at the LCLS as previously described^65^. Rhodopsin was solubilized in *n*-dodecyl-β-maltoside to a concentration of 8.4 mg ml^−1^ (0.2 mM). The samples were photoactivated using 480-nm laser pulses 50 fs in duration, focused through a 1/e^2^ spot of 100 μm in diameter (59 μm FWHM) with pulse powers of 6 μJ (110 mJ cm^−2^ averaged across the FWHM; 3,000 GW cm^−2^ peak power), 22 μJ (400 mJ cm^−2^ averaged across the FWHM; 11,200 GW cm^−2^ peak power), 45 μJ (830 mJ cm^−2^ averaged across the FWHM; 22,900 GW cm^−2^ peak power) and 89 μJ (1,640 mJ cm^−2^ averaged across the FWHM; 45,300 GW cm^−2^ peak power). Laser-induced heating was estimated from these data from the time delays of 10 ps ≤ Δ*t* ≤ 1 μs (Extended Data Fig. 3f), but the sparse sampling and signal-to-noise of these data did not allow multiple heating basis spectra to be extracted over this time domain. By contrast, TR-XSS studies on detergent solubilized photosynthetic reaction centre allowed two basis spectra to be extracted over the first 100 ps after photoexcitation. In that study, the amplitude associated with the first heating basis spectrum had reached its maximum value by Δ*t* = 10 ps, which then transitioned to a longer-timescale heating basis spectrum with the amplitudes of these two components crossing near Δ*t* = 40 ps (ref. ^65^). The principal singular value decomposition (SVD) component from our rhodopsin TR-XSS data was compared with temperature calibration curves (Extended Data Fig. 3g) to estimate the laser-induced change in temperature for different photoexcitation fluence, as described previously^65^. The heating impulse therefore imparted to the sample is summarized in Extended Data Fig. 3h, with a negative time point used as a control (plotted as zero laser fluence). Whereas these TR-XSS data indicate that laser-induced heating of detergent-solubilized samples of rhodopsin varies linearly with the pump laser pulse fluence, this should not be taken to imply that the photoexcitation is occurring in the single photon per chromophore linear response limit. Moreover, any energy stored within the protein on this time-scale (for example, as strain within the retinal) will not be visible as heating in the TR-XSS data, and therefore any measurable heating above a fraction of a photon equivalent implies that excess energy entered the system through a multiphoton absorption pathway.

Using Extended Data Fig. 3h as a laser-heating-induced calibration curve, we can estimate that the temperature jump induced in detergent solubilized rhodopsin samples injected using a GDVN liquid microjet exposed to the photoexcitation conditions used at SwissFEL (fluence of 140 mJ cm^−2^ averaged over the FWHM) would be Δ*T* = 0.016 ± 0.009 °C; and when exposed to the photoexcitations used at SACLA (fluence of 260 mJ cm^−2^ averaged over the FWHM) would be Δ*T* = 0.028 ± 0.016 °C. Using the formula for absorbed photons per molecule = Δ*T* × *C*_*P*_/([rhodopsin] × *A* × *hν*), where *C*_*P*_ = 3.8 J cm^−3^ is chosen as an approximate heat capacity for membrane proteins in solution with high detergent content (table 2 of ref. ^19^), *A* is Avogadro’s constant, *h* is Planck’s constant, *ν* = *c*/*λ* is the frequency of the pump laser photon, *c* is the speed of light and *λ* is the pump laser wavelength. These heating changes would correspond to an excess of 1.2 ± 0.7 photons absorbed by the rhodopsin chromophore under the photoexcitation conditions at SwissFEL, and an excess of 2.1 ± 1.2 photons under the photoexcitation conditions used at SACLA. However, considerable uncertainty must be acknowledged before extrapolating these estimates to our TR-SFX studies. Three of the most important considerations are that appropriate TR-XSS control studies should be performed using similar sample preparations for TR-XSS and TR-SFX studies as well as using the same microjet injector (that is, rhodopsin prepared in LCP and injected using a viscous injector); the above estimates assume that a good spatial overlap between the X-ray beam and laser focus was achieved and this did not drift substantially during the TR-XSS data collection at the LCLS; and ideally the same fs laser should be used for photoexcitation and the same tools should be used to measure the laser focus spot diameter in both TR-XSS and TR-SFX experiments. Despite the additional uncertainty arising from these shortcomings, the above estimates are notably lower than those calculated as *σF*/*hν* = 45 for the photoexcitation conditions used at SwissFEL and *σF*/*hν* = 81 for the photoexcitation conditions use at SACLA (*σ*_480 nm_ = 34,000 M^−1^ cm^−1^). There has been considerable debate about what constitutes appropriate photoexcitation conditions for TR-SFX studies^3,25,31,32^. Our TR-XSS observations suggest that, as with other TR-XSS studies on other light-sensitive proteins^3^, laser-induced sample heating is not accurately predicted by the product *σF*/*hν*. This may be due to the cross-section of the first excited state at 480 nm being much lower than that of the ground state; may be due to the photoexcited states having relatively high stimulated Raman scattering and stimulated emission cross-sections and consequently the absorbed excess energy is carried away by emitted photons rather than being visible as sample heating; there will be some energy loss due to scattering from the microjet; and there may be other factors that we do not yet understand. After adjusting for rhodopsin having a concentration of 4 mM in crystals but 0.2 mM in the above TR-XSS studies, these heating estimates imply that it is improbable that the temperature jump within crystals was the order of 100 °C as has been claimed for TR-SFX studies of bacteriorhodopsin^32^ It is also improbable that our TR-SFX data are dominated by the quasi-isotropic structural expansion due to laser-induced heating that was observed for a photosynthetic reaction centre in TR-XSS studies in which approximately 800 photons were absorbed per chromophore^65^.

### Data processing

All data were indexed using INDEXAMAJIG with the XGANDALF algorithm for data collected at SwissFEL (SF dark, 1 ps, 10 ps) and the MOSFLM^66^, DirAx^67^ and XGANDALF^68^ algorithms for data collected at SACLA (SACLA dark, 100 ps). The integration radius was set to 2 pixels for SwissFEL data and 3 pixels for SACLA data, while the background annulus was set to between 4 and 6 pixels for SwissFEL data and between 4 and 7 pixels for data collected at SACLA. The crystal-to-detector distance was optimized on a per-run basis by sampling detector distances between 91.5 mm and 97.5 mm (SwissFEL) and between 47.5 mm and 53.5 mm (SACLA) first at 200 µm and then 20-µm increments to determine the detector distance at which the standard deviations of the unit cell dimensions were minimized.

The SwissFEL and SACLA data were scaled and merged separately in PARTIALATOR^69^, using partiality modelling with XSPHERE^70^. Custom splitting was used to output separate dark and light-activated reflection files for data collected at each free electron laser.

A lattice translocation defect was identified in the crystals^36^ after inspection of the Patterson map with phenix.xtriage^71^. In particular, the Patterson peak at td = (0.000, 0.245, 0.000) (‘Translation vector (*Td* )’ in Extended Data Table 1 and Extended Data Fig. 1d–i) was attributed to the presence of two translation-related domains in the crystals. The correction^36^ required to retrieve single-domain intensities was described previously^72^, the percentage of molecules in the translated domain (*κ*) (‘Translated fraction (*k*)’ in Extended Data Table 1 and Extended Data Fig. 1d–i) was determined by correcting the intensities at increasing *κ* values from 0% to 50% in 1% increments until the (0.000, 0.245, 0.000) Patterson peak was flattened. The correction led to a reduction in the SACLA dark state Rfree from 26.11% to 23.92% and improved the interpretability of the dark-state electron density maps, which enabled further improvement of the model (Extended Data Fig. 1d–i).

### Structure determination and refinement of rhodopsin dark state

PDB 1U19 (ref. ^73^) with solvent and ligand molecules removed was used as a molecular replacement search model in Phaser MR^74^. The dark-state structure was obtained after several iterative cycles of refinement and iterative model building using Phenix.refine^75^ and Coot^76^. An additional ligand geometry file was generated using JLigand to restrain the geometry of the PSB linking the lysine side chain to retinal^77^.

### Calculation of difference density maps

*F*_o_(light) and *F*_o_(dark) amplitudes were calculated from the lattice translation defect corrected intensities using *phenix.french_wilson*^71^ and *F*_o_(light) − *F*_o_(dark) difference maps were calculated using phenix.fobs_minus_fobs_map^71^ using the multiscaling option excluding amplitudes smaller than 3*σ* and using reflections within the resolution range between 9 Å and 1.8 Å. All *F*_obs_(light) − *F*_obs_(dark) were computed using phases of the refined dark state.

For the calculation of *F*_calc_ − *F*_calc_ difference maps, the *F*_calc_ amplitudes were computed using SFall, scaled against experimental data using Scaleit and difference maps were calculated using FFT to a resolution of 1.7 Å, all programs were available in the CCP4 suite^78^.

For integrating and plotting density for the fluence response curve, the two 10-ps light datasets and the 1-ps light dataset recorded at SwissFEL were reduced to the size of the smallest dataset (about 29,000 patterns). These data were than scaled and merged as described for the map calculation, and difference maps were calculated in the exact same way. A custom MATLAB script based on ref. ^79^ was used to integrate the entire positive difference density in a 2 Å radius around the C_20_ and C_12_ atoms of the excited state. The density was then plotted against the fluence.

### Data extrapolation

Extrapolated data were calculated using the lattice translation corrected data and according to a method described previously^80^. A linear approximation was used as follows: *F*_extra_ = 100/*A* × (*F*_ob_(light) − *F*_obs_(dark)) + *F*_calc_, where *A* is the activation level in percent, *F*_extra_ represents the extrapolated structure factor amplitudes and *F*_calc_ represents the calculated amplitudes of the dark-state model. The activation level for each timepoint was determined independently using a previously described method^80^. In brief, extrapolated data were calculated with activation levels ranging from 10% to 50% in 1% increments, and 2*F*_extra_ − *F*_calc_ difference maps together with phases from the dark-state model were calculated. Negative 2*F*_extra_ − *F*_calc_ density around C_11_, C_12_ and C_20_ of retinal, which display negative density features in the *F*_obs_(light) − *F*_obs_(dark) maps, was integrated with a radius of 1.5 Å and above at 1.5*σ* cut-off for each activation level. Negative 2*F*_extra_ − *F*_calc_ difference density was plotted as a function of activation level; when the activation level is overestimated, there is little 2*F*_extra_ − *F*_calc_ negative difference density at these atomic positions, while the magnitude of the negative 2*F*_extra_ − *F*_calc_ difference density increases when the activation level is underestimated. The activation level is then determined by calculating the intersection between the two linear sections of the plot to find the activation level at which the negative density begins to appear. As the three light-activated datasets were collected under different experimental conditions, they were calculated independently (SwissFEL 1 ps 21%, SwissFEL 10 ps 20%, SACLA 100 ps 22%).

### Refinement of light-activated states

The dark-state model from SwissFEL was used as an initial model for refinement with the extrapolated data for the 1-ps and 10-ps timepoints in Phenix.refine^75^, interactive model building with Coot^76^ was performed to fit the model to the 2*F*_extra_ − *F*_calc_ maps and to remove water molecules lacking electron density. The same iterative procedure was performed starting with the SACLA 100-ps data, using the SACLA dark-state model as a starting point.

### Residue numbering

In addition to a number according to their position in the primary sequence, residues in rhodopsin are also assigned a ‘general’ number according to the Ballesteros–Weinstein scheme^81^. The Ballesteros–Weinstein general number consists of two numbers separated by a dot, where the first denotes the helix (1 to 8) and the second the position relative to the most-conserved residue in that helix, arbitrarily assigned to 50. For example, Glu113^3.28^ denotes that the counterion Glu113 is located in TM3 and twenty-two residues before the most conserved residue in TM3 (Arg135^3.50^).

The Ballesteros numbering for most of the amino acids in this study are Met44^1.39^, Met86^2.53^, Phe91^2.58^, Thr94^2.61^, Glu113^3.28^, Ala117^3.32^, Thr118^3.33^, Cys110^3.25^, Gly120^3.35^, Gly121^3.36^, Glu122^3.37^, Met207^5.42^, His211^5.46^, Phe212^5.47^, Pro215^5.50^, Phe261^6.44^, Trp265^6.48^, Pro267^6.50^, Tyr268^6.51^, Ala269^6.52^, Ala292^7.39^, Phe293^7.40^ and Lys296^7.43^.

#### Water nomenclature

W01 (chain C/HOH #01) at the tip of C_20_/RET; W02 (chain C/HOH #02) at Ser186; W03 (chain C/HOH #103) proximal to counterion Glu113 (Gly90, Phe91, Ala117); W04 (chain C/HOH #119) at Met86 (Phe91, Phe116, Ala117), low occupancy increasing by Δ*t* = 1 ps, resetting by Δ*t* = 100 ps.

### QM/MM calculations

The TR‐SFX crystallography structures reported in this work were used as initial geometry for the calculations. The pKa values at pH 9.0 of titratable amino acid residues in the protein were obtained using the PROPKA program^82,83^. Subsequently, the program tleap from the AMBER software package was used to protonate the protein by considering the previously calculated pKa values^84^ We performed first a short (50 steps) molecular mechanics (MM) energy minimization while applying positional restraints to the retinal and Lys296^7.43^ to relieve steric clashes. Subsequently, the geometries of both dark and batho states (1 ps and 100 ps) of rhodopsin were optimized using hybrid QM/MM^85^ in the gas phase. The backbone of the protein was kept frozen during the simulation. In the simplest system, the QM part consists of only retinal chromophore and the sidechain of Lys296 that forms the protonated Schiff base (RPSB). The hydrogen link atom (HLA) scheme^86^ was used to place the QM/MM boundary in between the C_δ_ and C_ε_ atoms of the Lys296 sidechain. We also considered one more extended QM region that includes the proximal counterion (Glu113^3.28^), ancestral counterion (Glu181^ECL2^), Ser186^ECL2^, Tyr191^ECL2^, Tyr268^6.51^ and water W01. The QM part was described using the BP86‐D3(BJ) functional^87,88^ in conjunction with the cc‐pVDZ basis set^89^ and the def2/J auxiliary basis set for the resolution of identity^90^. The Chain of Spheres exchange (COSX) algorithm was used in combination with the resolution of identity for the Coulomb term (RI‐J). The remaining protein was treated with the Amber ff14SB force field^91^. The TIP3P model was used to describe the water molecules^92^. The QM/MM optimizations were performed by using the quantum chemistry program Orca (v.5.0.2)^93^ interfaced with the DL_POLY module of the ChemShell (v.3.7.1) software package^94,95^. The minimized ground-state geometries and partial charges were used to calculate the vertical excitation energies at the RI‐ADC(2) level of theory^96^ with frozen core orbitals and cc‐pVTZ basis set in association with the corresponding auxiliary basis^89^. Moreover, to account for the effect of QM/MM geometry optimization on the excitation, we calculated the vertical excitation energies on the partially MM minimized structures of both dark and batho states for the simplest QM/MM system. The RI‐ADC(2) calculations were performed using the Turbomole (v.7.5.1) program package^97^. All of the calculations were performed using the supercomputing facilities at the Paul Scherrer Institute.

### Molecular dynamics simulations

We used molecular dynamics simulation data from the GPCRmd database^98^, an open access research resource that hosts a comprehensive dataset of molecular dynamics simulations for most GPCR 3D structures solved to date. The GPCRmd offers several tools to analyse simulation trajectories interactively or, alternatively, they can be downloaded and analysed locally. Specifically, we concatenated three simulation replicas (3 × 2,500 frames) of rhodopsin (PDB: 1GZM; trajectory IDs 16414, 16415 and 16416) embedded into a lipid bilayer solvated with water and ions and simulated for an aggregated time of 1.5 µs (that is, 500 ns per replica). We used Python (v.3.10) and the MDanalysis library^99,100^ to fetch the simulation data and to compute the average root mean square fluctuation of the protein Cα atoms.

### Reporting summary

Further information on research design is available in the Nature Portfolio Reporting Summary linked to this article.

## Online content

Any methods, additional references, Nature Portfolio reporting summaries, source data, extended data, supplementary information, acknowledgements, peer review information; details of author contributions and competing interests; and statements of data and code availability are available at 10.1038/s41586-023-05863-6.

### Supplementary information

Reporting Summary

Supplementary Video 1 Time-lapse GIF image of the retinal-binding pocket after 0 ps, 1  ps and 100 ps of rhodopsin photoactivation. The three models of rhodopsin in the dark (red) and at 1 ps (yellow) and 100 ps (green) of photoactivation are superimposed. The video shows a time-lapse GIF of the retinal-binding pocket of those models contoured successively with the electron density of the dark (2*F*_o_ − *F*_c_), then the 1 ps (2*F*_ext_ − *F*_c_), then 100 ps (2*F*_ext_ − *F*_c_) maps. The many weak interactions are shown as dashed lines, and yellow arrows point to the regions changing the most.

## Figures and Tables

**Extended Data Table 1:**
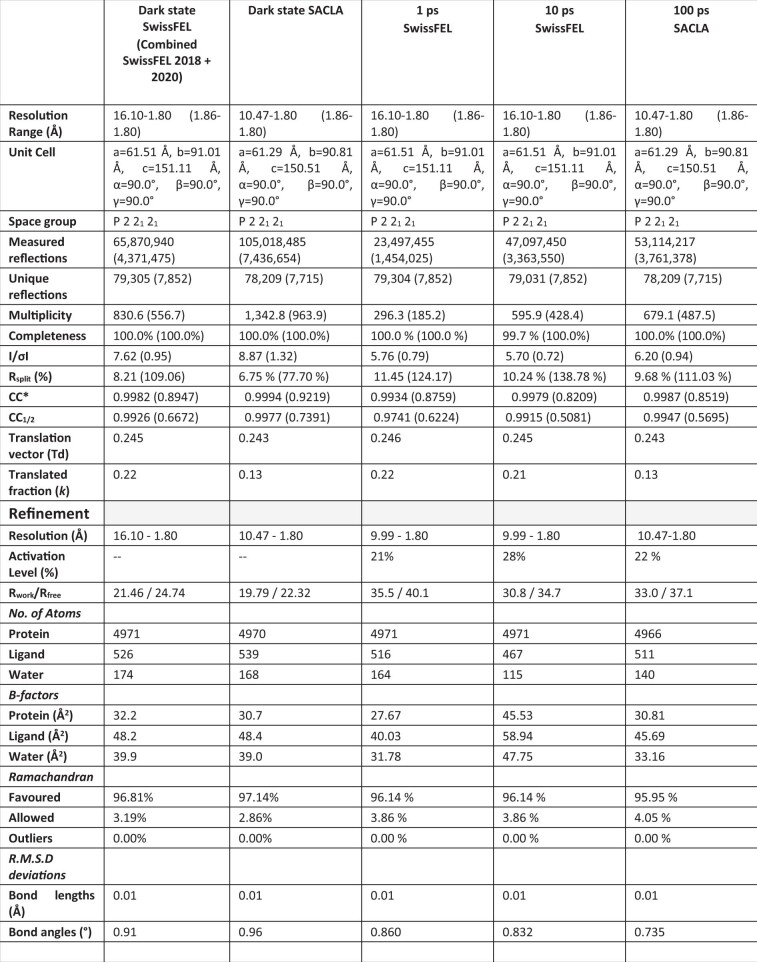
Crystallographic data statistics for rhodopsin structures after 1, 10 and 100 picoseconds of photoactivation, compared to the dark state

**Extended Data Table 2:**
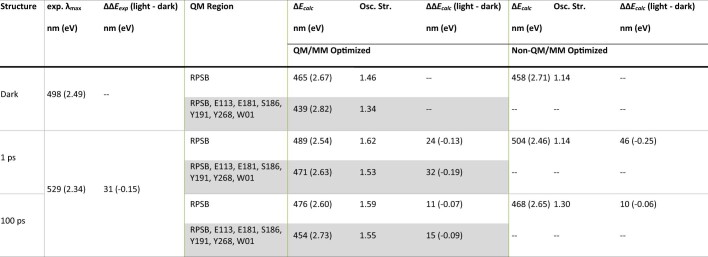
Vertical excitation energies computed by quantum mechanics/molecular mechanics (QM/MM) calculation

Vertical excitation energies (Δ*E*_*calc*_) and oscillator strengths for dark, 1 ps, and 100 ps photoactivated rhodopsin. The excitation energies were calculated on both QM/MM optimized and non-optimized geometries. In case of QM/MM optimized geometries, two QM regions were considered: a) retinal protonated Schiff base (RPSB), and b) retinal PSB including key nearby polar residues and a water molecule (RPSB, E113, E181, S186, Y191, Y268, W01). The values were computed with the RI‐ADC(2) method using the cc-pVTZ basis set. The experimental values for the absorption maxima of each state (exp. λ_max_) are provided for comparison. In case of the 1 ps intermediate, we observe a difference of 22 nm in the calculated red-shifts between the QM/MM optimized and no-optimized structures, which can be attributed to the small structural rearrangement triggered due to geometry optimization near retinal PSB region. For 100 ps, the red-shifts of the optimized and non-optimized structures are comparable, but considerably smaller than the 1 ps rhodopsin red-shift.

**Extended Data Table 3:**
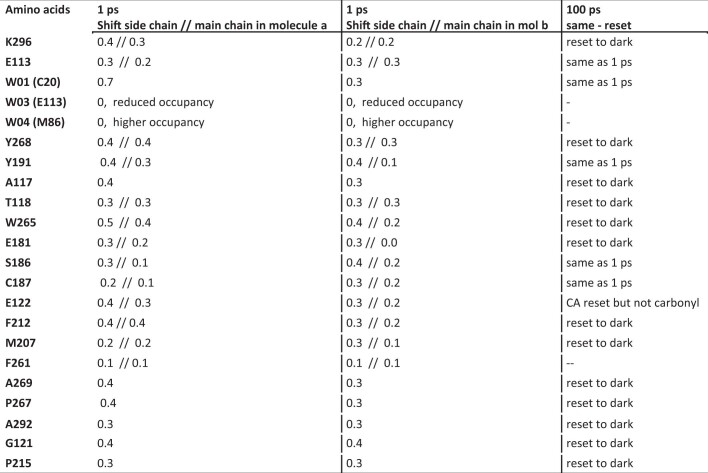
Amplitude of amino acids shifts (Å) after 1ps photoactivation

Rhodopsin structures from the 1 ps and 100 ps time-delay of photoactivation datasets were compared to the dark state structure obtained by SFX. A list of the amino acids changing of position (**first column**) is related to the amplitude of the local shift (in Å) at the side chain and main chain of molecules a (**second column**) or b (**third column**) of the asymmetric unit after 1 ps. The **fourth column** documents if the displacement after 1 ps of photoactivation reset or persist after 100 ps. Remarkable changes of water occupancy at the same position are mentioned. The ancestral counterion is E181 (ref. ^8^) and Y191 and Y268 are part of its surrounding tyrosine cage^105^.

## Data Availability

Coordinates and structure factors have been deposited at the PDB under accession codes 7ZBC (rhodopsin in the dark state obtained by SFX at the SACLA), 7ZBE (rhodopsin in the dark state obtained by SFX at the SwissFEL), 8A6C (rhodopsin after 1 ps photoactivation obtained by TR-SFX at the SwissFEL), 8A6D (rhodopsin after 10 ps photoactivation obtained by TR-SFX at the SwissFEL) and 8A6E (rhodopsin after 100 ps photoactivation obtained by TR-SFX at the SACLA).

## Code availability

The difference electron density maps (*F*_o(light, 1, 10 and 100 ps time delays)_ − *F*_o(dark)_) of mammalian rhodopsin photoactivated for 1, 10 and 100 ps, respectively, are available at the Swiss National Supercomputing Center CSCS repository (10.16907/577a2a74-22ea-4d01-87af-6a99447a430f). The codes for the lattice translation disorder correction and the calculation of the electron density extrapolated maps are available at Zenodo (10.5281/zenodo.7560364).
