# Supplementary material for: Ultrafast structural changes direct the first molecular events of vision
Source: Nature. 2023 Mar 22;615(7954):939–44. doi: 10.1038/s41586-023-05863-6 (PMC10060157; doi:10.1038/s41586-023-05863-6)
Supplement: Supplementary file 1 — Reporting Summary [file 41586_2023_5863_MOESM1_ESM.pdf]

nature portfolio

Corresponding author(s): Gebhard Schertler & Valerie Panneels

Last updated by author(s): Feb 8, 2023

Reporting Summary

Nature Portfolio wishes to improve the reproducibility of the work that we publish. This form provides structure for consistency and transparency in reporting. For further information on Nature Portfolio policies, see our Editorial Policies and the Editorial Policy Checklist.

Statistics

For all statistical analyses, confirm that the following items are present in the figure legend, table legend, main text, or Methods section.

n/a	Confirmed	
☒	☐	The exact sample size (n) for each experimental group/condition, given as a discrete number and unit of measurement
☒	☐	A statement on whether measurements were taken from distinct samples or whether the same sample was measured repeatedly
☒	☐	The statistical test(s) used AND whether they are one- or two-sided<br>*Only common tests should be described solely by name; describe more complex techniques in the Methods section.*
☒	☐	A description of all covariates tested
☒	☐	A description of any assumptions or corrections, such as tests of normality and adjustment for multiple comparisons
☐	☒	A full description of the statistical parameters including central tendency (e.g. means) or other basic estimates (e.g. regression coefficient) AND variation (e.g. standard deviation) or associated estimates of uncertainty (e.g. confidence intervals)
☒	☐	For null hypothesis testing, the test statistic (e.g. F, t, r) with confidence intervals, effect sizes, degrees of freedom and P value noted<br>*Give P values as exact values whenever suitable.*
☒	☐	For Bayesian analysis, information on the choice of priors and Markov chain Monte Carlo settings
☒	☐	For hierarchical and complex designs, identification of the appropriate level for tests and full reporting of outcomes
☒	☐	Estimates of effect sizes (e.g. Cohen's d, Pearson's r), indicating how they were calculated

Our web collection on statistics for biologists contains articles on many of the points above.

Software and code

Policy information about availability of computer code

Data collection	TR-SFX SETTINGS: wxPython at the SACLA (doi: 10.1107/S1600576716005720) and Python 3.8 at the SwissFEL.<br>PREPROCESSING ON THE FLY: Peakfinder8 from Cheetah 2018.05 (doi:10.1107/S1600576714007626) and CrystFEL 0.9.1 (doi:10.1107/S0021889812002312)
Data analysis	CRYSTALLOGRAPHY: CrystFEL 0.9.1 (doi:10.1107/S0021889812002312), Phenix 1.19.2-4158 (doi: 10.1107/S2059798319011471), CCP4i 7.1 (doi:10.1107/S0907444910045749), Coot-0.9.6 (doi: 10.1107/S0907444904019158)<br>CODE FOR LATTICE TRANSLATION CORRECTION: Zenodo repository under the link with doi: https://doi.org/10.5281/zenodo.7560364.<br>CODE FOR CALCULATION OF EXTRAPOLATED MAPS: doi: 10.1038/s41592-019-0628 and https://doi.org/10.5281/zenodo.7560364.<br>QM/MM: PROPKA 3.4.0; AmberTools21; ORCA 5.0.2; ChemShell 3.7.1; TURBOMOLE 7.5.1

For manuscripts utilizing custom algorithms or software that are central to the research but not yet described in published literature, software must be made available to editors and reviewers. We strongly encourage code deposition in a community repository (e.g. GitHub). See the Nature Portfolio guidelines for submitting code & software for further information.

Data

Policy information about availability of data

All manuscripts must include a data availability statement. This statement should provide the following information, where applicable:

- Accession codes, unique identifiers, or web links for publicly available datasets
- A description of any restrictions on data availability
- For clinical datasets or third party data, please ensure that the statement adheres to our policy

Coordinates and structure factors have been deposited in the Protein Data Bank under accession codes 7ZBC (SFX dark state rhodopsin at the SACLA), 7ZBE (SFX

dark state rhodopsin at the SACLA). Ready for submission: time-delays 1ps, 10ps and 100ps.Difference electron density maps are available at the doi: doi.psi.ch/detail/10.16907/577a2a74-22ea-4d01-87af-6a99447a430f

Field-specific reporting

Please select the one below that is the best fit for your research. If you are not sure, read the appropriate sections before making your selection.

☒ Life sciences ☐ Behavioural & social sciences ☐ Ecological, evolutionary & environmental sciences

For a reference copy of the document with all sections, see nature.com/documents/nr-reporting-summary-flat.pdf

Life sciences study design

All studies must disclose on these points even when the disclosure is negative.

Sample size	Methods part mentions that the amount of collected X-ray diffraction frames was of about 30'000, which gives a quality of the electron density maps requested for observing ultrafast small amplitude changes inside the protein and obtaining the good statistics detailed in the Table 1.
Data exclusions	no data has been excluded.
Replication	while replication of an XFEL beamtime is an utopy, we state in the main text (Line 128) that our room temperature SFX-data of rhodopsin in the dark state at the SACLA XFEL and SwissFEL are reproducible because "very similar to other crystal structures collected at cryogenic temperatures (e.g., 1GZM; RMSD = 0.33 Å on Cα atoms)". For the picosecond-illuminated rhodopsin datasets, the Extended Figure 5 shows the replication and reproducibility of the difference electron density detected in two different beamtimes (SwissFEL 2018 and 2020) and at two different laser power.
Randomization	The robustness of serial crystallographic data is confirmed using the statistic comparison of random half datasets. Models and maps are typically not analyzed using randomization. However robust tools are available to ensure the correctness of the structures and maps obtained.
Blinding	As is typical in macromolecular crystallography experiments, the observed reflections comprising each dataset were partitioned into a working set and a test set. The models were refined against the working sets, while the test sets were not used. After refinement, the agreement between the model and the working set are calculated (Rwork), as well as the agreement between the model and the test set (Rfree). A significantly lower Rwork than Rfree indicates that the subjective building of the model into the electron density maps may suffer from overfitting. The use of the Rfree as an unbiased indicator of the information content of crystallographic models has been described in detail previously (Brunger, Nature, 1992, vol 395).

Reporting for specific materials, systems and methods

We require information from authors about some types of materials, experimental systems and methods used in many studies. Here, indicate whether each material, system or method listed is relevant to your study. If you are not sure if a list item applies to your research, read the appropriate section before selecting a response.

Materials & experimental systems

n/a	Involved in the study
☒	☐ Antibodies
☒	☐ Eukaryotic cell lines
☒	☐ Palaeontology and archaeology
☒	☐ Animals and other organisms
☒	☐ Human research participants
☒	☐ Clinical data
☒	☐ Dual use research of concern

Methods

n/a	Involved in the study
☒	☐ ChIP-seq
☒	☐ Flow cytometry
☒	☐ MRI-based neuroimaging
